# BPG: Seamless, automated and interactive visualization of scientific data

**DOI:** 10.1186/s12859-019-2610-2

**Published:** 2019-01-21

**Authors:** Christine P’ng, Jeffrey Green, Lauren C. Chong, Daryl Waggott, Stephenie D. Prokopec, Mehrdad Shamsi, Francis Nguyen, Denise Y. F. Mak, Felix Lam, Marco A. Albuquerque, Ying Wu, Esther H. Jung, Maud H. W. Starmans, Michelle A. Chan-Seng-Yue, Cindy Q. Yao, Bianca Liang, Emilie Lalonde, Syed Haider, Nicole A. Simone, Dorota Sendorek, Kenneth C. Chu, Nathalie C. Moon, Natalie S. Fox, Michal R. Grzadkowski, Nicholas J. Harding, Clement Fung, Amanda R. Murdoch, Kathleen E. Houlahan, Jianxin Wang, David R. Garcia, Richard de Borja, Ren X. Sun, Xihui Lin, Gregory M. Chen, Aileen Lu, Yu-Jia Shiah, Amin Zia, Ryan Kearns, Paul C. Boutros

**Affiliations:** 1 Ontario Institute for Cancer Research, Toronto, Canada; 2 Department of Medical Biophysics, University of Toronto, Toronto, Canada; 3 Department of Pharmacology and Toxicology, University of Toronto, Toronto, Canada; 4 Present address: Center for Computational Research, Buffalo Institute for Genomics and Data Analytics, NYS Center for Excellence in Bioinformatics & Life Science, University at Buffalo, Buffalo, USA; 5 Department of Human Genetics, University of California, Los Angeles, USA; 6 Department of Urology, University of California, Los Angeles, USA; 7 Institute for Precision Health, University of California, Los Angeles, USA; 8 Jonsson Comprehensive Cancer Center, University of California, Los Angeles, USA

**Keywords:** Data-visualization, Interactive plotting, Software, Web-resources

## Abstract

**Background:**

We introduce BPG, a framework for generating publication-quality, highly-customizable plots in the R statistical environment.

**Results:**

This open-source package includes multiple methods of displaying high-dimensional datasets and facilitates generation of complex multi-panel figures, making it suitable for complex datasets. A web-based interactive tool allows online figure customization, from which R code can be downloaded for integration with computational pipelines.

**Conclusion:**

BPG provides a new approach for linking interactive and scripted data visualization and is available at http://labs.oicr.on.ca/boutros-lab/software/bpg or via CRAN at https://cran.r-project.org/web/packages/BoutrosLab.plotting.general

**Electronic supplementary material:**

The online version of this article (10.1186/s12859-019-2610-2) contains supplementary material, which is available to authorized users.

## Background

Biological experiments are increasingly generating large, multifaceted datasets. Exploring such data and communicating observations is, in turn, growing more difficult and the need for robust scientific data-visualization is accelerating [1–4]. Myriad data visualization tools exist, particularly as web-based interfaces and local software packages. Unfortunately these often do not integrate easily into R-based statistical pipelines, such as the widely used Bioconductor [5]. Within R, many visualization packages exist, including base graphics [6], ggplot2 [7], lattice [8], Sushi [9], circlize [10], multiDimBio [11], NetBioV [12], GenomeGraphs [13] and ggbio [14]. There is also a broad range of activity-specific visualization packages focused on specific tasks or analysis-types [15–24]. Some of these lack publication-quality defaults such as high-resolution, appropriate label-sizing and default colour palettes appropriate for gray-scale use and visible for those with red-green colour-blindness. Many can require significant parameterization. Others contain limited plot types, provide limited scope for automatic generation of multi-panel figures or are constrained to specific data-types. Few allow interactive visualization, where specific plot elements can be highlighted and the set of parameters available to customize them automatically identified and allowing interactive generation of R code through a GUI interface that visualizes plot changes in real-time. Thus while each of these visualization packages has significant value and user-bases, each lacks some features beneficial for computational biologists and data scientists.

Good visualization software must create a wide variety of chart-types in order to match the diversity of data-types available. It should provide flexible parametrization for highly customized figures and allow for multiple output formats while employing reasonable, publication-appropriate default settings, such as producing high resolution output. In addition, it should integrate seamlessly with existing computational pipelines while also providing an easily intuitive, interactive mode. There should be an ability to transition between pipeline and interactive mode, allowing cyclical development. Finally, good design principles should be encouraged, such as suggesting appropriate color choices and layouts for specific use-cases. To help users quickly gain proficiency, detailed examples, tutorials, an ability for real-time interactive plot-tuning and an application programming interface (API) are required. To date, no existing visualization suite fully fills these needs.

## Implementation

**Fig. 1:**
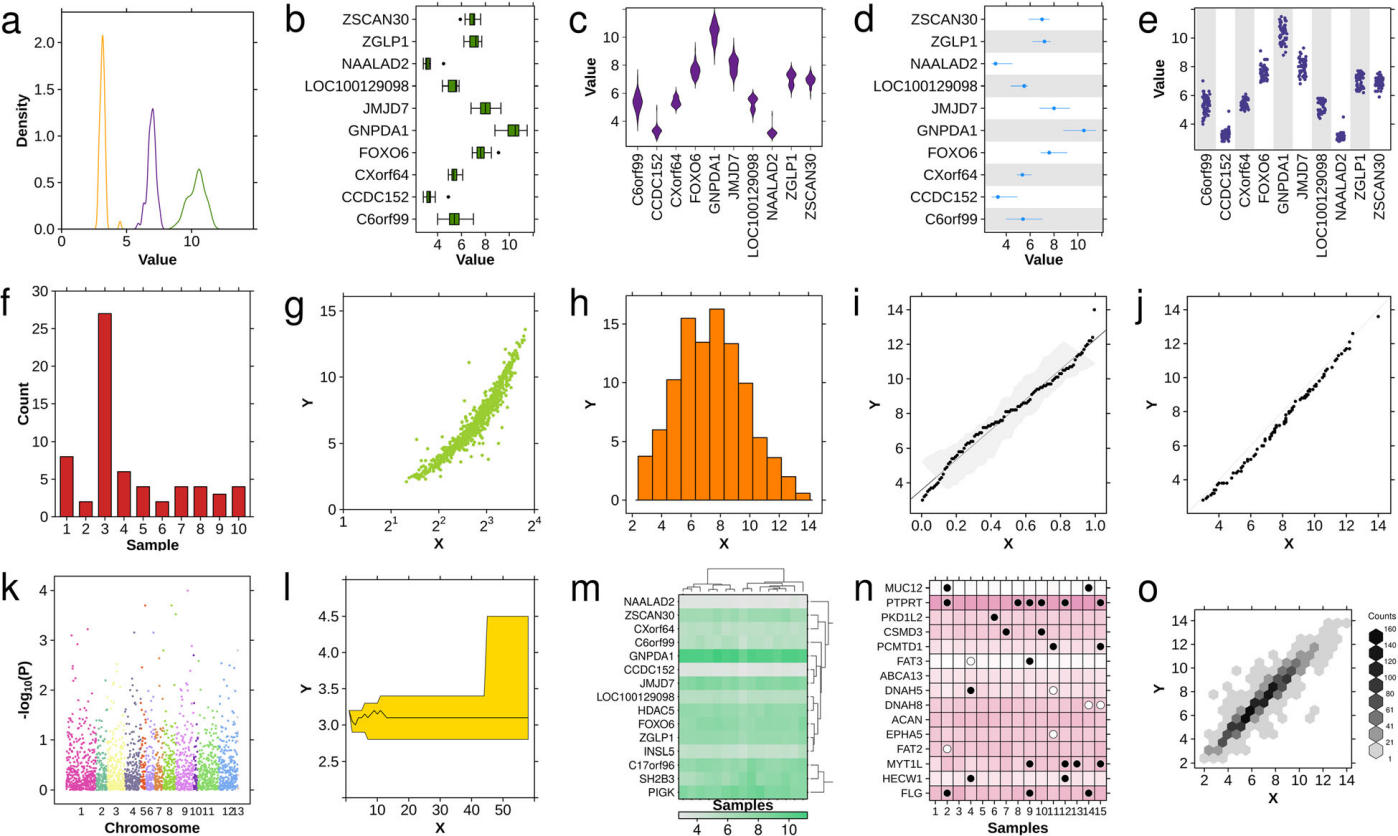
Available chart-types. The basic chart-types available in BPG: **a** density plot, **b** boxplot, **c** violin plot, **d** segplot, **e** strip plot, **f** barplot, **g** scatterplot, **h** histogram, **i** qqplot fit, **j** qqplot comparison, **k** Manhattan plot, **l** polygon plot, **m** heatmap, **n** dotmap and **o** hexbinplot. All plots are based upon the datasets included in the BPG package and code is given in Additional file 1

To address this gap, we have created the BPG (BoutrosLab.plotting.general) library, which is implemented in R using the grid graphics system and lattice framework. It generates a broad suite of chart-types, ranging from common plots such as bar charts and box plots to more specialized plots, such as Manhattan plots (Fig. 1**;** code is in Additional file 1). These include some novel plot-types, including the dotmap: a grid of circles inset inside a matrix, allowing representation of four-dimensional data (Fig. 1n). Each plotting function is highly parameterized, allowing precise control over plot aesthetics. The default parameters for BPG produce high resolution (1600 dpi) TIFF files, appropriate for publication. The file type is specified simply by specifying a file extension. Other default values contribute to graphical consistency including: the inclusion of tick marks, selection of fonts and default colors that work together to create a consistent plotting style across a project. Default values have been optimized to generate high-quality figures, reducing the need for manual tuning. However, even good defaults will not be appropriate for every use-case [15]. Additional file 2: Figure S1 demonstrates a single scatter plot created using four separate graphics frameworks with either default or optimized settings: BPG, base R graphics, ggplot2, and lattice. BPG required half as much code as the other frameworks for both default and optimized plots, while producing plots with at least similar quality (Additional file 3).

To facilitate rapid graphical prototyping, an online interactive plotting interface was created (http://bpg.oicr.on.ca). This interface allows users to easily and rapidly see the results of adjusting parameter values, thereby encouraging precise improvement of plot aesthetics. The R code generated by this interface is also made available for download, as is a methods paragraph allowing careful reporting of plotting options. A public web-interface is available, and local interfaces can be easily created.

**Fig. 2:**
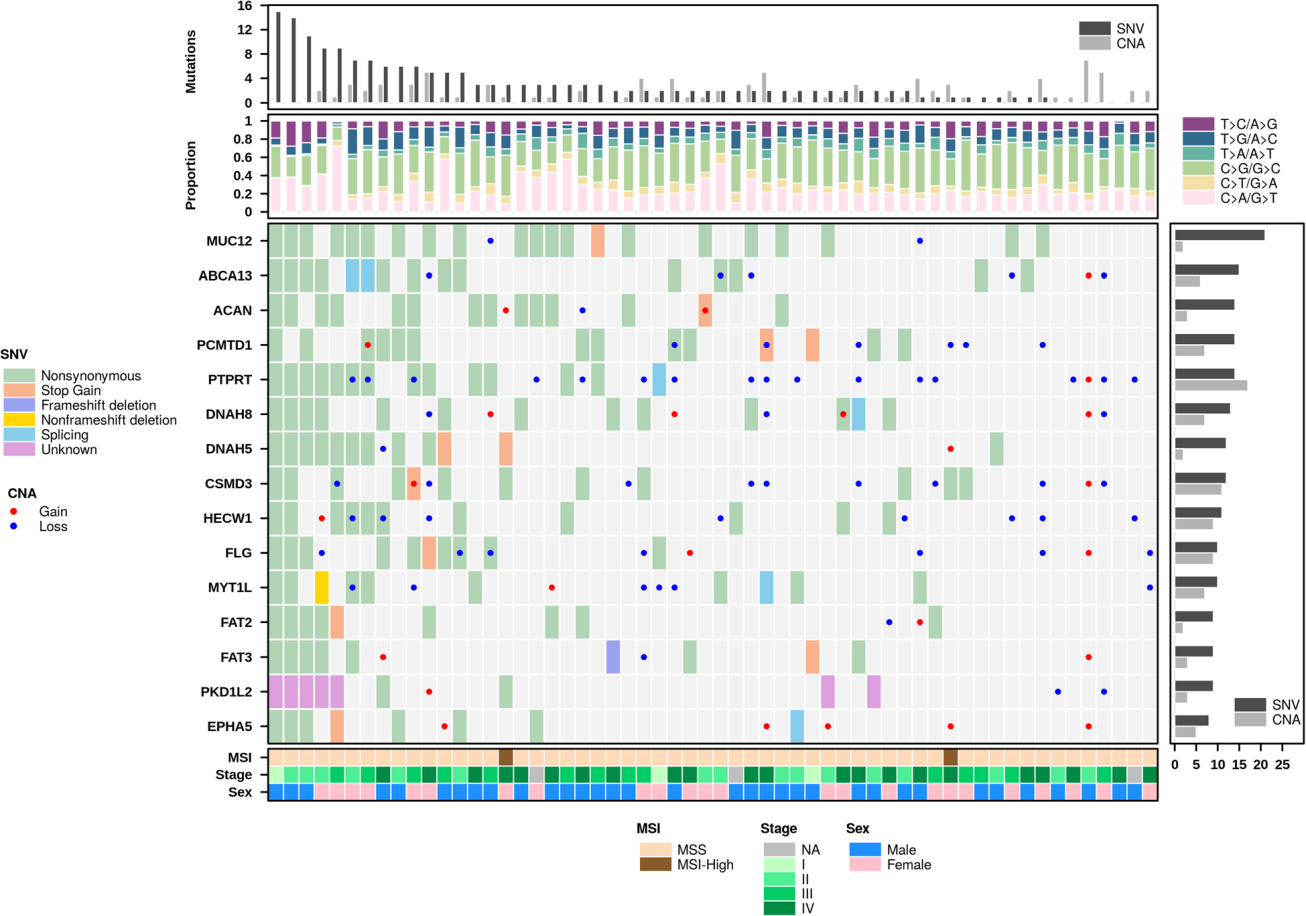
Multiplot example. The create.multiplot function is able to join multiple chart-types together into a single figure. In this example, a central dotmap conveys the somatic mutations present in a selection of genes (y-axis) for a number of colorectal tumours (x-axis), while adjacent barplots and heatmaps provide additional information. Within this central dotmap, shaded cells reflect single nucleotide variants (SNVs), while dots in cells reflect copy number aberration (CNAs), which some patients have both types of aberration in a single gene (shaded cells harbouring a dot). The colour of the cell or dot indicates the specific type of mutation, using the legend on the left. The bottom heatmap shows key clinical information about each patient, including their Sex, the Stage of their disease and their microsatellite status (instable, MSI; stable MSS). The barplot to the right shows the percentage of patients with a SNV or a CNA in that gene. The barplot at the top, equivalently, shows the number of SNVs and CNAs for each patient. Finally, the second barplot from the top categorizes all SNVs based on the type of base-change that mutation reflects, showing their proportion as a fraction of the total mutation number. Code used to generate this figure is available in Additional file 4

One critical feature of BPG is its ability to combine multiple plots into a single figure: a technique used widely in publications. This is accomplished by the create.multiplot function, which automatically aligns plots and standardizes parameters such as line widths and font sizes across all plot elements within the final figure. This replaces the slow and error-prone manual combination of figures using PowerPoint, LaTeX or other similar software, or the time-consuming parameterization of manually align plot locations directly in R with functions like layout(). The necessity of combining multiple plots arises from the complexity of datasets – with high dimensional data, it is often difficult to convey all relevant information within a single chart-type. Combining multiple chart-types allows more in depth visualization of the data. For example, one plot might convey the number of mutations present in different samples; a second plot could add the proportion of different mutation types, while a third could give sample-level information (Fig. 2). We have included a series of example datasets directly in BPG, including the one used to create the visualization in Fig. 2, and the source-code for creating this plot from these datasets is given in Additional file 4.

A number of utility functions in BPG assist in plot optimization, such as producing legends and covariate bars, or formatting text with scientific notation for *p*-values. One difficult step in creating figures is the selection of color schemes that are both pleasing and interpretable [25, 26]. BPG provides a suite of 45 color palettes including qualitative, sequential, and diverging color schemes [27], shown in Additional file 5: Figure S2. Many optimized color schemes exist for numerous use cases including tissue types, chromosomes and mutation types. The default.colors function produces a warning when a requested color scheme is not grey-scale compatible, a common concern for figures reproduced in black and white. This is determined by converting each color to a grey value between 1 and 100, and indicating differences of < 10 as not grey-scale compatible to approximate a color scheme’s visibility when printed in grey-scale. To facilitate reproducibility, image metadata is automatically generated for all plots, creating descriptors such as software and operating system versions.

## Results

Extensive documentation is provided to help new users learn how to use BPG. To assist researchers in determining which chart-type is appropriate for their dataset, we provide plotting examples in the documentation which are derived from a real dataset and a plotting guide is included to explain the intended use-case of each function. This guide also contains explanations of typography, basic color theory and layout design which help to improve the design of figures [28, 29]. In addition, an online API is available with both simple and complex use-case examples for each plot-type to help users quickly learn the range of functionality available.

## Conclusions

BPG has been used in over 60 publications to date (Additional file 6: Table S1) [30–35]. These plotting functions have been integrated into numerous R analysis pipelines for automated figure generation as part of the analysis of large –omic data. The plots created by this package are reproducible and maintain a consistent aesthetic. We believe that BPG will facilitate improved visualization and communication of complex datasets.

## Additional files

Additional file 1: Code to generate Fig. 1. (TXT 14 kb)

Additional file 2: **Figure S1.** Comparison of graphical software options in R. (a-b) are created with base R graphics, (c-d) are created using ggplot2, (e-f) are made in lattice and (g-h) use BPG. The first plot in each pair uses default settings, while the second plot has been adjusted for font sizes, axes ranges, tick mark locations, grid lines, diagonal lines, background shading and highlighted datapoints. The number of lines of code used to create default plots are: 10 for base R, 10 for ggplot2, 14 for lattice, and 5 for BPG. The customized plots use 73 lines for base R, 83 for ggplot2, 86 for lattice, and 42 for BPG. Code for generating this figure is provided in Additional file 3. (TIFF 1590 kb)

Additional file 3: Code to generate Additional file 2: Figure S1. (TXT 6 kb)

Additional file 4: Code to generate Fig. 2. (TXT 11 kb)

Additional file 5: **Figure S2.** Color palettes. Color palettes are provided using the default.colors function for (a) generic use-cases and force.color.scheme for (b) specific use-cases. This display is generated using the show.available.palettes function. Interactive display of colors is also available using the display.colors function. (TIF 1036 kb)

Additional file 6: **Table S1.** Publications using BPG. (DOC 67 kb)

## Abbreviations

API: Application Programming Interface
BPG: BoutrosLab.Plotting.General
CNA: Copy Number Aberration
CRAN: Comprehensive R Archive Network
DPI: Dots per Inch
GUI: Graphical User Interface
MSI: Microsatellite Instable
MSS: Microsatellite Stable
SNV: Single Nucleotide Variant

## References

[1] Grinstein G, Trutschl M, Cvek U. Proceedings of the visual data mining workshop. KDD. 2001:7–19.

[2] Anscombe FJ. Graphs in Statistical Analysis. Am Stat. 1973;27:17–21.

[3] Shoresh N, Wong B. Data exploration. Nat Methods. 2012;9:5. 10.1038/nmeth.1829 22312636

[4] O'Donoghue SI, et al. Visualizing biological data-now and in the future. Nat Methods. 2010;7:S2–4. 10.1038/nmeth.f.301 20195254

[5] Gentleman RC, et al. Bioconductor: open software development for computational biology and bioinformatics. Genome Biol. 2004;5:R80. 10.1186/gb-2004-5-10-r80 PMC545600 15461798

[6] R Core Team. R: A language and environment for statistical computing. R Foundation for Statistical Computing, Vienna, Austria. 2014. http://www.R-project.org/. Accessed 10 Jan 2019.

[7] Wickham, H. ggplot2: elegant graphics for data analysis. Springer, New York, USA, 2009.

[8] Sarkar, D. Lattice: multivariate data visualization with R. Springer, New York, USA, 2008.

[9] Phanstiel DH, Boyle AP, Araya CL, Snyder MP. Sushi R: flexible, quantitative and integrative genomic visualizations for publication-quality multi-panel figures. Bioinformatics. 2014;30:2808–10. 10.1093/bioinformatics/btu379 PMC4173017 24903420

[10] Gu Z, Gu L, Eils R, Schlesner M, Brors B. circlize Implements and enhances circular visualization in R. Bioinformatics. 2014;30:2811–2. 10.1093/bioinformatics/btu393 24930139

[11] Scarpino SV, Gillette R, Crews D. (R package). 2013. http://cran.r-project.org/web/packages/multiDimBio/index.html. Accessed 10 Jan 2019.

[12] Tripathi S, Dehmer M, Emmert-Streib F. NetBioV: an R package for visualizing large network data in biology and medicine. Bioinformatics. 2014;30:2834–6. 10.1093/bioinformatics/btu384 24928209

[13] Durinck S, Bullard J, Spellman PT, Dudoi S. GenomeGraphs: integrated genomic data visualization with R. BMC Bioinformatics. 2009;10:2. 10.1186/1471-2105-10-2 PMC2629762 19123956

[14] Yin T, Cook D, Lawrence M. ggbio: an R package for extending the grammar of graphics for genomic data. Genome Biol. 2012;13:R77. 10.1186/gb-2012-13-8-r77 PMC4053745 22937822

[15] He W, Zhao S, Zhang C, Vincent MS, Zhang B. QuickRNASeq: Guide for Pipeline Implementation and for Interactive Results Visualization. Methods Mol Biol. 2018;1751:57–70. 10.1007/978-1-4939-7710-9_4 29508289

[16] Waggott D, Chu K, Yin S, Wouters BG, Liu FF, Boutros PC. NanoStringNorm: an extensible R package for the pre-processing of NanoString mRNA and miRNA data. Bioinformatics. 2012;28(11):1546–8. 10.1093/bioinformatics/bts188 PMC3356845 22513995

[17] Sendorek DH, Lalonde E, Yao CQ, Sabelnykova VY, Bristow RG, Boutros PC. NanoStringNormCNV: pre-processing of NanoString CNV data. Bioinformatics. 2018;34(6):1034–6. 10.1093/bioinformatics/btx707 PMC5860631 29112706

[18] Ranjitha Dhanasekaran A, Gardiner KJ. RPPAware: A software suite to preprocess, analyze and visualize reverse phase protein array data. J Bioinform Comput Biol. 1850001 (2018). 10.1142/S0219720018500014 29478376

[19] Lee TR, Ahn JM, Kim G, Kim S. IVAG: An Integrative Visualization Application for Various Types of Genomic Data Based on R-Shiny and the Docker Platform. Genomics Inform. 2017;15(4):178–82. 10.5808/GI.2017.15.4.178 PMC5769861 29307145

[20] Renault V, Tost J, Pichon F, Wang-Renault SF, Letouzé E, Imbeaud S, Zucman-Rossi J, Deleuze JF, How-Kit A. aCNViewer: Comprehensive genome-wide visualization of absolute copy number and copy neutral variations. PLoS One. 2017;12(12):e0189334. 10.1371/journal.pone.0189334 PMC5736239 29261730

[21] Zhu X, Wolfgruber TK, Tasato A, Arisdakessian C, Garmire DG, Garmire LX. Granatum: a graphical single-cell RNA-Seq analysis pipeline for genomics scientists. Genome Med. 2017;9(1):108. 10.1186/s13073-017-0492-3 PMC5716224 29202807

[22] Jalili V, Matteucci M, Masseroli M. Ceri S. Explorative visual analytics on interval-based genomic data and their metadata. BMC Bioinformatics. 2017;18(1):536. 10.1186/s12859-017-1945-9 PMC5715631 29202689

[23] Turner D, Sutton JM, Reynolds DM, Sim EM, Petty NK. Visualization of Phage Genomic Data: Comparative Genomics and Publication-Quality Diagrams. Methods Mol Biol. 2018;1681:239–60. 10.1007/978-1-4939-7343-9_18 29134600

[24] Li J, Akbani R, Zhao W, Lu Y, Weinstein JN, Mills GB, Liang H. Explore, Visualize, and Analyze Functional Cancer Proteomic Data Using the Cancer Proteome Atlas. Cancer Res. 2017;77(21):e51–4. 10.1158/0008-5472.CAN-17-0369 PMC5679242 29092939

[25] Rougier NP, Droettboom M, Bourne PE. Ten Simple Rules for Better Figures. PLoS Comput Biol. 2014;10:e1003833. 10.1371/journal.pcbi.1003833 PMC4161295 25210732

[26] Wong B. Color coding. Nat Methods. 2010;7:573. 10.1038/nmeth0810-573 20704014

[27] Wong B. Color blindness. Nat Methods. 2011;8:441. 10.1038/nmeth.1618 21774112

[28] Harrower H, Brewer CA. ColorBrewer.org: An Online Tool for Selecting Colour Schemes for Maps. Cartogr J. 2003;40:27–37.

[29] Wong B. Points of review (part 1). Nat Methods. 2011;8:101. 10.1038/nmeth0211-101 21355115

[30] Haider S, et al. Pathway-based subnetworks enable cross-disease biomarker discovery. Nat Commun. 2018;9(1):4746. 10.1038/s41467-018-07021-3 PMC6232113 30420699

[31] Lee AY, et al. Combining accurate tumor genome simulation with crowdsourcing to benchmark somatic structural variant detection. Genome Biol. 2018;19(1):188. 10.1186/s13059-018-1539-5 PMC6219177 30400818

[32] Espiritu SMG, et al. The Evolutionary Landscape of Localized Prostate Cancers Drives Clinical Aggression. Cell. 2018;173(4):1003–13. 10.1016/j.cell.2018.03.029 29681457

[33] Fraser M, et al. Genomic hallmarks of localized, non-indolent prostate cancer. Nature. 2017;541(7637):359–64. 10.1038/nature20788 28068672

[34] Boutros PC, et al. Spatial genomic heterogeneity within localized, multifocal prostate cancer. Nat Genet. 2015;47(7):736–45. 10.1038/ng.3315 26005866

[35] Ewing AD, et al. Combining tumor genome simulation with crowdsourcing to benchmark somatic single-nucleotide-variant detection. Nat Meth. 2015;12:623–30. 10.1038/nmeth.3407 PMC4856034 25984700

