# Supplementary figures and images for: BPG: Seamless, automated and interactive visualization of scientific data
Source: BMC Bioinformatics. 2019 Jan 21;20:42. doi: 10.1186/s12859-019-2610-2 (PMC6341661; doi:10.1186/s12859-019-2610-2)

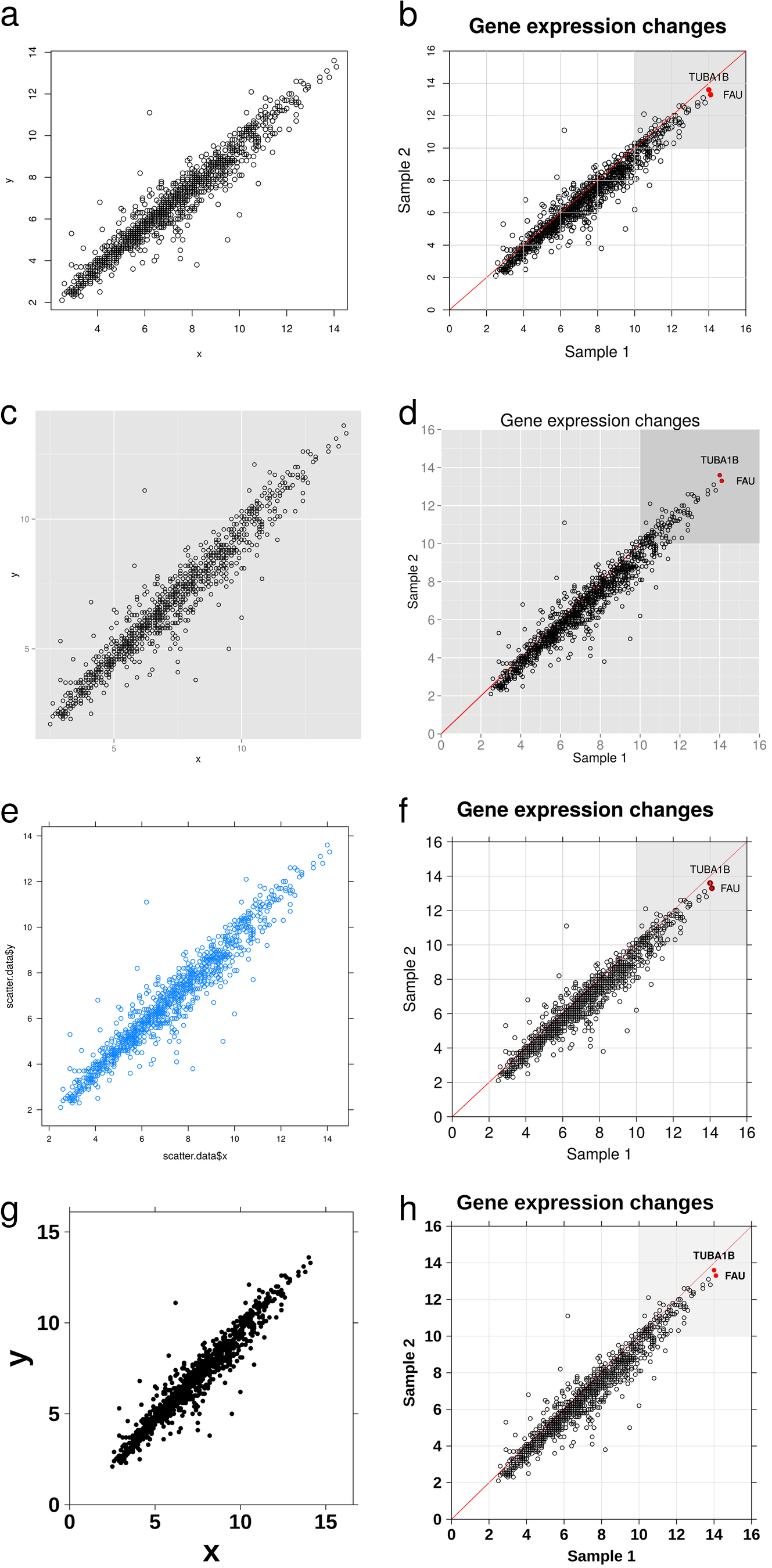

Supplement: Supplementary file 2 — Figure S1. Comparison of graphical software options in R. (a-b) are created with base R graphics, (c-d) are created using ggplot2, (e-f) are made in lattice and (g-h) use BPG. The first plot in each pair uses default settings, while the second plot has been adjusted for font sizes, axes ranges, tick mark locations, grid lines, diagonal lines, background shading and highlighted datapoints. The number of lines of code used to create default plots are: 10 for base R, 10 for ggplot2, 14 for lattice, and 5 for BPG. The customized plots use 73 lines for base R, 83 for ggplot2, 86 for lattice, and 42 for BPG. Code for generating this figure is provided in Additional file 3. (TIFF 1590 kb) [file 12859_2019_2610_MOESM2_ESM.tiff]

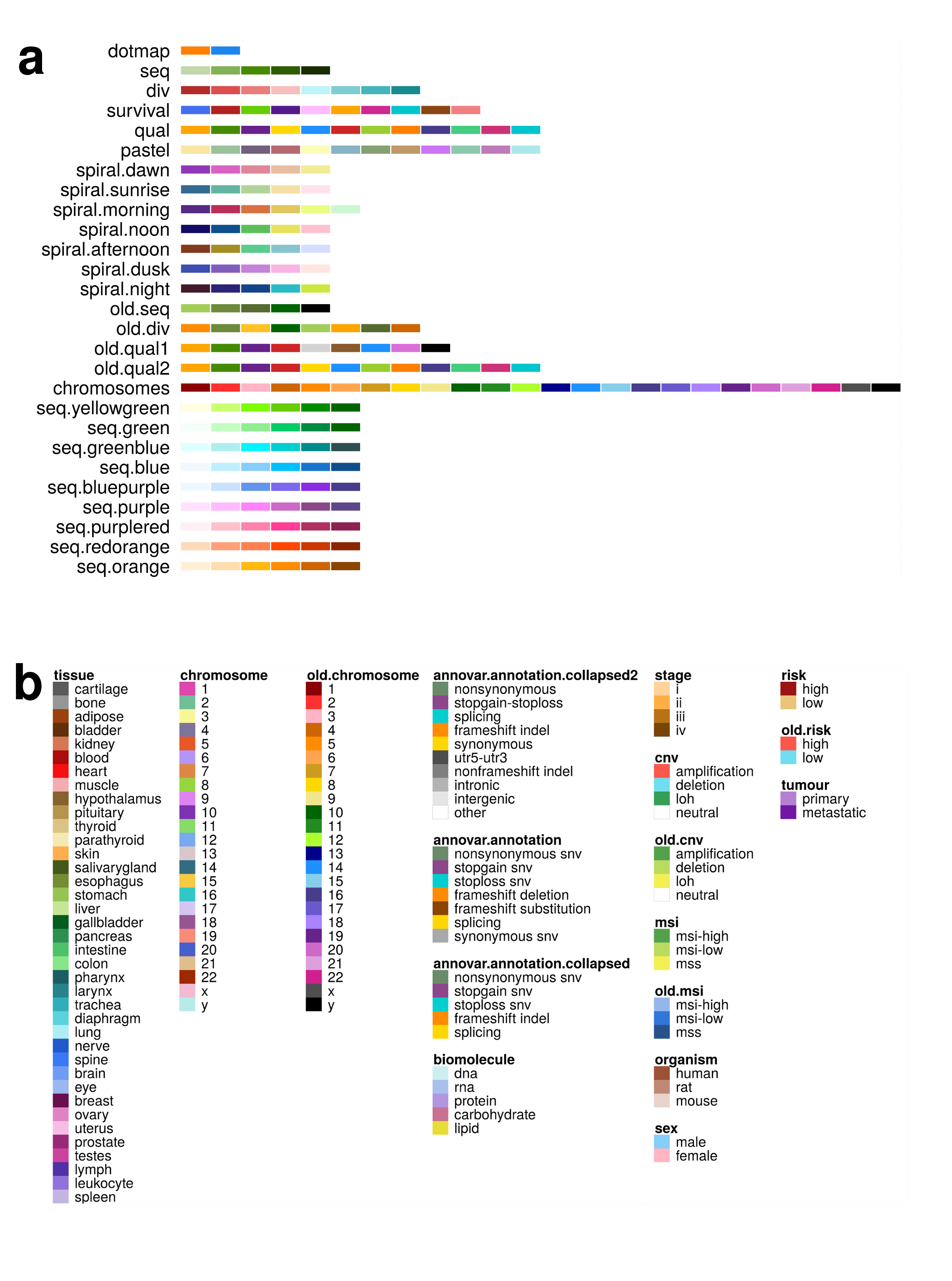

Supplement: Supplementary file 5 — Figure S2. Color palettes. Color palettes are provided using the default.colors function for (a) generic use-cases and force.color.scheme for (b) specific use-cases. This display is generated using the show.available.palettes function. Interactive display of colors is also available using the display.colors function. (TIF 1036 kb) [file 12859_2019_2610_MOESM5_ESM.tif]
