# Supplementary material for: BPG: Seamless, automated and interactive visualization of scientific data
Source: BMC Bioinformatics. 2019 Jan 21;20:42. doi: 10.1186/s12859-019-2610-2 (PMC6341661; doi:10.1186/s12859-019-2610-2)
Supplement: Supplementary file 6 — Table S1. Publications using BPG. (DOC 67 kb) [file 12859_2019_2610_MOESM6_ESM.doc]

**Supplementary Table 1. Selected Publications using BPG**

| **Year** | **First author** | **Journal** | **Issue** | **PMID** |
| --- | --- | --- | --- | --- |
| 2018 | Haider, S. | Nature Communications | 9(1):4746 | 30420699 |
| 2018 | Kalkat, M. | Molecular Cell | In press | 30415952 |
| 2018 | Lee, A.Y. | Genome Biology | 19(1):188 | 30400818 |
| 2018 | Grzadkowski, M.R. | BMC Bioinformatics | 19(1):400 | 30390622 |
| 2018 | Tu, W.B. | Cancer Cell | 34(4):579-595 | 30300580 |
| 2018 | Li, C.H. | Cancer Research | 78(19):5527-5537 | 30275052 |
| 2018 | Cooper, C.I. | BMC Bioinformatics | 19(1):339 | 30253747 |
| 2018 | Gong, I.Y. | PLoS One | 13(9):e0204123 | 30216362 |
| 2018 | Casey, A.E. | Journal of Cell Biology | 217(8):2951-2974 | 29921600 |
| 2018 | Wither, J.E. | PLoS One | 13(5):e0196117 | 29742110 |
| 2018 | Espiritu, S.M.G. | Cell | 173(4):1003-1013 | 29681457 |
| 2018 | Sendorek, D.H. | BMC Bioinformatics | 19(1):28 | 29385983 |
| 2018 | Hopkins, J.F. | Gastroenterology | 154(6):1620-1624 | 29378198 |
| 2017 | Sendorek, D.H. | Bioinformatics | 34(6):1034-1036 | 29112706 |
| 2017 | Hopkins, J.F. | Nat Comm | 8(1):656 | 28939825 |
| 2017 | Alfaro, J.A. | Genome Med | 9(1):62 | 28716134 |
| 2017 | Shiah, Y.J. | Bioinformatics | 33(20):3151-3157 | 28605401 |
| 2017 | Chua, M.L.K. | Eur Urol | 72(5):665-674 | 28511883 |
| 2017 | Briollais, L. | J Natl Cancer Inst | 109(4) | 28376164 |
| 2017 | Thapa, B. | J Thorac Oncol | 12(5):850-859 | 28257959 |
| 2017 | Prokopec, S.D. | BMC Genomics | 18(1):78 | 28086803 |
| 2017 | Fraser, M. | Nature | 541(7637):359-364 | 28068672 |
| 2017 | Taylor, R.A. | Nat Commun | 8:13671 | 28067867 |
| 2017 | Lalonde, E. | Eur Urol | 72(1):22-31 | 27815082 |
| 2016 | Huang, B.F. | BMC Bioinformatics | 17(1):331 | 27586051 |
| 2016 | Kim, Y. | Nat Commun | 7:11906 | 27350604 |
| 2016 | Bhandari, V. | Genomics | 108(2):78-83 | 27311755 |
| 2016 | Watson, J.D. | Arch Toxicol | 91(1):325-338 | 27136898 |
| 2016 | Gagliano, S.A. | Am J Hum Genet | 98(5):956-62 | 27087318 |
| 2015 | Lee, J. | BMC Genomics | 16(1):625 | 26290441 |
| 2015 | Huang, X. | Oncotarget | 6(26):22439-22451 | 26068950 |
| 2015 | Koulahan, K.E. | Toxicol Appl Pharmicol | 288(2):223-231 | 26232522 |
| 2015 | Boutros, P.C. | Nature Genetics | 47(7):736-745 | 26005866 |
| 2015 | Ewing, A.D. | Nature Methods | 12, 623-630 | 25984700 |
| 2015 | Anghel, C.V. | BMC Bioinformatics | 16(1): 156 | 25972088 |
| 2015 | Dodbiba, L. | PLoS ONE | 10(3): e0121872 | 25826681 |
| 2015 | Prokopec, S.D. | Toxicol Appl Pharmicol | 284(2): 188-196 | 25703434 |
| 2015 | Shiah, Y.J. | Stem Cell Reports | 4(3):313-22 | 25702641 |
| 2015 | Starmans, M.H. | Clinical Cancer Research | 21(6):1477-86 | 25609067 |
| 2015 | Houlahan, K.E. | Toxicology | 328:93-101 | 25529477 |
| 2015 | Yao, C.Q. | Cancer Medicine | 4(1): 56-64 | 25314936 |
| 2014 | Sun, R.X. | BMC Genomics | 15:1053 | 25467400 |
| 2014 | Lalonde, E. | Lancet Oncology | 15(13):1521-32 | 25456371 |
| 2014 | Berlin, A. | Oncotarget | 5(22):11081-90 | 25415046 |
| 2014 | van den Beucken, T. | Nature Communications | 5:5203 | 25351418 |
| 2014 | Prokopec, S.D. | PLoS ONE | 9(10):e110730 | 25329058 |
| 2014 | Sepiashvili, L. | Mol Cell Proteomics | 13(12): 3572-84 | 25271301 |
| 2014 | Chong, L.C | Nature Methods | 11(10):1071-5 | 25173705 |
| 2014 | Fox, N.S. | BMC Bioinformatics | 15:170 | 24902696 |
| 2014 | Goard, C.A. | Breast Cancer Res Treat | 143(2):301-12 | 24337703 |
| 2014 | Cappello, P. | Oncogene | 33(18):2375-84 | 23708664 |
| 2014 | Govind, S.K. | BMC Bioinformatics | 15:78 | 24646301 |
| 2014 | Hoeben, B.A.W. | BMC Cancer | 14:130 | 24571588 |
| 2014 | Watson, J.D. | Toxicol Appl Pharm | 274:445-54 | 24355419 |
| 2013 | Koritzinsky, M. | J Cell Biol | 203(4):615-27 | 24247433 |
| 2013 | Liew, M.S. | Cancer Medicine | 2(6):916-24 | 24403265 |
| 2013 | Wasylishen, A.R. | Cancer Research | 73(21):6504-15 | 24030976 |
| 2013 | John, T. | PloS ONE | 8(7):e67876 | 23935846 |
| 2013 | Prokopec, S.D. | Chem-Biol Interact | 205:63-71 | 23791969 |
| 2013 | Nichols, A.C. | Case Rep Oncol Med | ID 270362 | 23653877 |
| 2013 | Prokopec, S.D. | RNA | 19:51-62 | 23169800 |
| 2012 | Kim, Y. | Mol Cell Proteomics | 11(12):1870-84 | 22986220 |
| 2012 | Lipina, T.V. | PLoS ONE | 7(12):e51562 | 23272119 |
| 2012 | Starmans, M.H. | Genome Medicine | 4:84 | 23146350 |
| 2012 | Starmans, M.H. | British Journal of Cancer | 107:508-15 | 22722312 |
| 2012 | Cortese, R. | Human Molecular Genetics | 21(16):3619-31 | 22619380 |
| 2012 | Starmans, M.H. | Radiotherapy & Oncology | 102:436-43 | 22356756 |
| 2012 | Yao, C.Q. | Toxicol Appl Pharm | 260:135-45 | 22342509 |
| 2012 | Ng, T. | Regulatory Peptides | 174:79-89 | 22209827 |
| 2011 | Boutros, P.C. | PLoS ONE | 6(7):e18337 | 21760882 |
| 2011 | Yan, R. | Bioinformatics | 27(15):2054-61 | 21685048 |
| 2011 | Elschenbroich, S. | J Proteome Research | 10:2286-99 | 21491939 |
| 2011 | Reich, H.N. | J Molecular Diagnostics | 13(2):143-51 | 21354048 |
| 2011 | Boutros, P.C. | Toxicol Appl Pharm | 251:119-29 | 21215274 |
| 2010 | Boutros, P.C. | Cancer Informatics | 9:197-208 | 20838609 |
| 2010 | Wong, W.W. | BMC Cancer | 10:351 | 20598143 |
| 2010 | Moffat, I.D. | BMC Genomics | 11:263 | 20420666 |
| 2009 | Boutros, P.C. | Toxicological Sciences | 112(1):245-56 | 19759094 |
| 2009 | Hui, A.B. | Laboratory Investigation | 89(5):597-606 | 19290006 |
| 2009 | Boutros, P.C. | Proc Natl Acad Sci | 106(8):2624-8 | 19196983 |
